# Efficacy of ultrasound versus short wave diathermy in the treatment of chronic low back pain in patients with lumbar disk herniation: a prospective randomized control study

**DOI:** 10.1186/s13102-023-00769-2

**Published:** 2023-11-20

**Authors:** Selin Ozen, Sukran Guzel, Huma Boluk Senlikci, Sacide Nur Saracgil Cosar, Ebru Selin Selcuk

**Affiliations:** 1 Faculty of Medicine, Department of Physical Medicine and Rehabilitation, Baskent University, Ankara, Turkey; 2 Ankara Etlik City Hospital, Physical Medicine and Rehabilitation Hospital, Ankara, Turkey; 3 Ankara City Hospital, Physical Medicine and Rehabilitation Hospital, Ankara, Turkey; 4 Department of Physical Medicine and Rehabilitation, Dr.Abdurrahman Yurtaslan Ankara Oncology Education and Research Hospital, Ankara, Turkey

**Keywords:** Diathermy, Low back pain, Physical therapy modalities, Physical therapy specialty, Ultrasonography

## Abstract

**Background:**

Lumbar disk herniation (LDH) is a cause of chronic low back pain (CLBP) treated using physical therapy (PT), including exercise and physical modalities such as ultrasound (US) and short wave diathermy (SWD). Despite the use of US and SWD, there is inconclusive evidence on their efficacy. The aim of this study was to investigate the efficacy of US and SWD in the treatment of CLBP in patients with LDH.

**Methods:**

A prospective randomized control clinical study. Individuals with radicular CLBP and LDH on magnetic resonance imaging, presenting to the Physical and Rehabilitation Medicine Department were randomized into 3 treatment groups. All participants received 10 sessions of hotpack, transcutaneous nerve stimulation (TENS) and therapeutic exercises. In addition, Group 1 received 10 sessions of therapeutic US (1 MHz, 1.5W/cm2, 10 min), Group 2 SWD (27.12 MHz, wavelength 11.06 m, induction technique, 20 min) to the lower back. Group 3 (control group) received hotpack, TENS and therapeutic exercises alone. Visual analogue scale (VAS) for LBP, Modified Oswestry Disability Index (MODI) and Short Form 36 (SF-36) were evaluated pre and post treatment and at one and three months follow up.

**Results:**

In all groups, VAS for LBP and MODI improved with treatment and at the one and three month follow up (*p* < 0.001). In Groups 1 and 2, MODI scores continued to reduce at 1 and 3 months (*p* < 0.001 and *p* = 0.012 respectively). SF-36 physical, social function and pain parameters reduced in all groups (*p* < 0.05). Role limitation due to physical and emotional problems, emotional well-being, vitality and mental health improved in Groups 1 and 2 (*p* < 0.05).

**Conclusions:**

Deep heating agents can be used as part of the physical therapy for CLBP in those with LDH with positive mid-term effects.

**Trial registration:**

NCT03835182, 02/04/2019.

## Introduction

Chronic low back pain (CLBP) can be defined as low back pain lasting for longer than three months in the lumbar spine region and has a reported prevalence of up to 25.4% in those between 20 and 59 years old [1]. Chronic low back pain limits functionality and activities of daily living (ADL) as well as increasing healthcare expenditure both directly and indirectly [2]. Herniation of the intervertebral disk into the spinal canal is a cause of radicular low back pain (LBP) resulting in nerve or dorsal root ganglion irritation and/or compression and consequent radicular leg pain in a dermatomal distribution [3].

The conservative treatment of CLBP includes physical therapy (PT), namely exercise and physical modalities, which aim to reduce pain and improve functionality [4, 5]. Physical agents most frequently used as part of the treatment of CLBP include hot pack, electrotherapy and deep-heating agents [6, 7]. It is believed that the thermal and mechanical effects of deep heating modalities, which include therapeutic ultrasound (US) and short wave diathermy (SWD), results in an increase in blood flow and metabolism and enhancement of collagen extensibility, reduction in connective tissue stiffness and muscle spasm [8–10].

Studies on the use of PT modalities such as US in the treatment of lumbar disc herniation (LDH) have suggested that they may have beneficial effects on LBP and disability [11] however there is a paucity of evidence on this subject. Overall, the benefits of US and SWD in the treatment of LBP are still uncertain [12, 13]. The aim of this study was to investigate the efficacy of US and SWD in the treatment of CLBP in patients with LDH.

## Methods

A prospective randomized control study conducted between September 2018 and December 2021. A total of 330 patients between the ages of 20 and 60 presenting to Baskent University Faculty of Medicine, Department of Physical and Rehabilitation Medicine (PRM) with LBP of at least three months duration and physical examination and magnetic resonance imaging consistent with a diagnosis of LDH + radiculopathy [14] were screened for study inclusion. Exclusion criteria were: 1) Presence of another mechanical aetiology of LBP (scoliosis, spondylolithesis, compression fracture of the vertebra) 2) Presence of a non-mechanical aetiology of LBP (infection, seronegative spondyloarthropathy, metabolic bone disease) 3) History of spinal surgery 4) History of PT for LBP over the past six months 5) Use of regular pharmacological agents for pain relief 6) Pregnancy 7) Presence of metallic prosthesis/ cardiac pacemaker 8) Contraindication to heat exposure/ heat intolerance.

### Group allocation

One hundred and one patients presenting to the PRM departments throughout the duration of the study, met the inclusion criteria. Randomization and group allocation was performed by a clinician who had no other involvement in the study. Patient age, sex, body mass index (BMI), LBP duration and stage of disc protrusion based on MRI findings were recorded.

### Interventions

All patients received a total of ten one-hourly sessions of PT on an inpatient basis over a two week period; five consecutive sessions on five consecutive days per week. All treatments were administered by a single physiotherapist. All patients included in the study received a one-to-one supervised therapeutic exercise program once per day, lasting twenty minutes, intergrated into each treatment session consisting of posture, neck, upper and lower back range of motion and progressive resistance exercises and hamstring stretching individualised to the patients’ needs. In addition, individuals allocated to Group 1 received hot pack (HP, 71–74°c, 20 min duration), transcutaneous nerve stimulation (TENS) (Enraf Nonius TensMed P82) frequency 100 Hz, pulse duration 20-60 ms (biphasic), 100 pulse per second for 20 minutes (min) duration via four 5 × 5 cm self-adhesive surface electrodes) and therapeutic US (Enraf Nonius Sonopuls) 490, frequency 1 MHz, 1.5W/cm^2^, 10 min duration) applied to the lower back. Individuals allocated to Group 2 received continous SWD (cSWD) (Curapuls 419), frequency 27.12 MHz, wavelength 11.06 m, induction technique, 20 min duration) alongside the same HP and TENS treatment applied to the lower back. Individuals allocated to Group 3 (control group) received HP and TENS treatment only. During the inpatient stay and follow up period of the study, participants with inadequate pain control were administered paracetamol at a maximum dose of 1 g four times daily to aid pain relief and reminded that no other pharmacological analgesics were allowed for the duration of the study.

### Outcome measures

All patients were evaluated before treatment, at the end of the ten sessions of PT, and at one and three months following treatment by a PRM specialist blind to the patients’ treatment group. The primary outcome measure was back pain. Study participants were assessed using:

1. Visual Analog Scale (VAS) for low back pain severity: A subjective visual pain score from 0 cm (no pain) to 10 cm [15].
2. Turkish version of the Modified Oswestry Disability Index (MODI): An outcome measure used to evaluate disability due to low back pain which consists of ten questions, including back pain severity and impact on ADL [16, 17]. Total scores range from 0–50 which are used to calculate the index percentage. A higher percentage signifies greater level of disability. A minimal detectable change of less than 10% maybe attributed to measurement error.
3. Turkish version of the Short form 36 (SF 36): A health survey used to assess health concepts including physical function and pain [18, 19]. Sub scales also assess: vitality, social functioning, role limitation due to emotional and physical problems and mental health. Higher scores signify improved well being. The 'transformed scale’ provides the final score; Transformed Scale = [(raw score- lowest possible raw score)/possible raw score range]100.

The study power was determined based on the study by Ansari et al. [20] and calculated using the Power and Sample Size Statistical Programme 3.1.2 (Vanderbilt University, 2015). Thirty one individuals per treatment group and 20 controls were recommended for a study power of 80% with a 5% type 1 error.

**Fig. 1:**
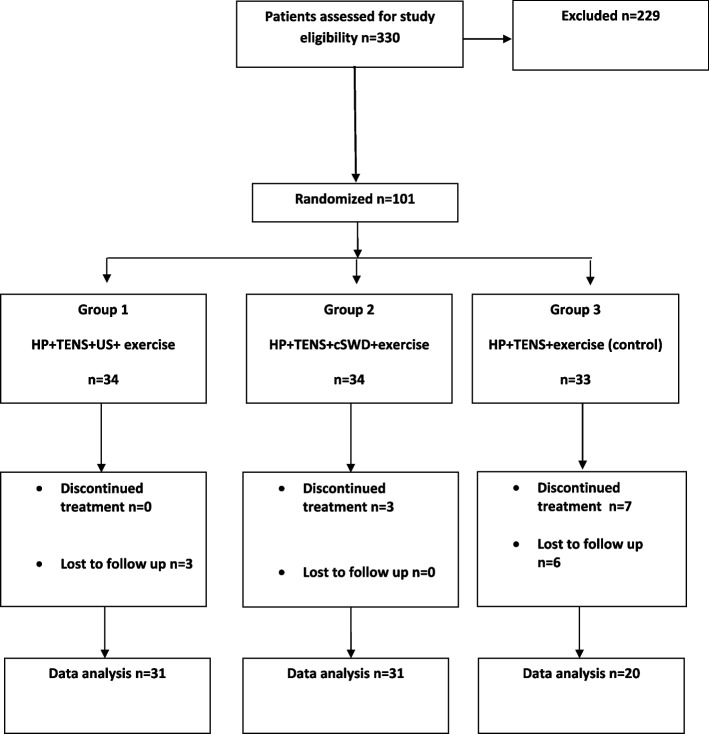
CONSORT diagram representing study participant flow through the study

The study participants were block randomized into three different treatment groups using The Random Allocation Software Program 1.0 (M. Saghaei, MD., Isfahan, Iran) (Fig. 1) [21].

All study involving human participants were in accordance with the ethical standards of the institutional research committee and with the 1964 Helsinki Declaration and its later amendments or comparable ethical standards. The study was approved by the Bioethics Committee of the Medical University of Baskent (Project No. KA13/236) and registered with ClinicalTrials.gov, trial number NCT03835182, initial registration date 02/04/2019. The contents of this study adhere to the CONSORT reporting guidelines [22].

### Statistical analysis

Statistical Package for Social Sciences (SPSS) version 22.0 (IBM Corporation, Armonk, NY, USA) was used for analysis of date. Means ± standard deviations and or medians of continuous variables and numbers and percentage of categorical variables were calculated. Kolmogorov Smirnov test was used to evaluate the normal distribution of continuous variables. Intergroup comparison of normally distributed qualitative variables was determined using the One way ANOVA (post hoc Bonferroni) and repeated measures ANOVA (Post hoc: paired sample T test) for within group analysis. Comparison of non normally distributed intergroup data was performed using the Kruskal Wallis (Bonferroni corrected Mann Whitney U test) and Friedman test (posthoc: Wilcoxon Signed Ranks test) for within group analysis. Categorical data was compared using the Chi square test. Spearman Correlation test was used for correlation between variables. *p* < 0.05, *p* < 0.016 for Bonferroni corrected analysis and *p* < 0.012 for paired sample T test represented statistical significance.

## Results

A total of 82 patients were included and completed the study between September 2018 and December 2021. Group 1 and 2 included 31 study participants and Group 3 consisted of 20 individuals (Fig. 1). The mean age of the patients was 48.85 ± 11.54 (*p* = 0.085) years with an average duration of symptoms of 12 (range 3–360) months. The most common level of disc herniation was L4-5 (*n* = 39, 47.6%) and disc protrusion was present in 75 (91.4%) patients (Table 1).

**Table 1:** Baseline characteristics of study participants

	**US group** ***n* = 31**	**KDD group** ***n* = 31**	**Control group** ***n* = 20**	**All patients** ***n* = 82**	***p***
**Mean age (years ± SD)**	45.25 ± 11.08	51.38 ± 11.21	50.50 ± 11.86	48.85 ± 11.54	0.085^*^
**Sex (M/F)**	10/21	8/23	8/12	26/56	0.566^**^
**BMI (mean ± SD)**	28.16 ± 4.81	28.49 ± 4.09	28.41 ± 5.25	28.34 ± 4.62	0.961^*^
**Symptom duration (months)** **Median (min–max)**	12 (3–360)	12 (3–300)	36 (3–120)	12 (3–360)	0.204^***^
**Anatomical location of disc herniation (n)**					
** L1-2 (n,%)**	2 (6.5)	4 (12.9)	3 (15)	9 (11)	0.541^**^
** L2-3 (n,%)**	5 (16.1)	4 (12.9)	0 (0)	9 (11)	0.180^**^
** L3-4 (n,%)**	7 (22.6)	6 (19.4)	2 (10)	15 (18.3)	0.578^**^
** L4-5 (n,%)**	14 (45.2)	18 (58.1)	7 (35)	39 (47.6)	0.403^**^
** L5-S1 (n,%)**	11 (35.5)	13 (41.9)	10 (50)	34 (41.5)	0.435^**^
**Stage of disc herniation (n,%)**					
** Protrusion**	27 (90)	28 (90.3)	20 (100.0)	75 (91.4)	0.482^**^
** Ekstrusion**	4 (12.9)	2 (6.5)	0	6 (7.3)	
** Sequestration**	0	1 (3.2)	0	1 (1.3)	
**Pre treatment VAS score ± SD**	7.19 ± 1.60	6.51 ± 1.72	6.70 ± 1.49	6.81 ± 1.63	0.249^*^
**Pre treatment Modifiye Oswestry score ± SD**	48.48 ± 18.20	50.19 ± 13.35	40.90 ± 17.16	47.28 ± 16.48	0.127^*^
** Pre treatment SF 36**					
** Physical functioning**	45.74 ± 21.26	47.09 ± 20.72	59.00 ± 27.65	49.48 ± 23.14	0.104^*^
** Role -physical**	0 (0–100)^a^	0 (0–100)^a^	87.5 (0–100)^a^	10 (0–100)	0.002^***^
** Role -emotional**	33 (0–100)^a^	33 (0–100^)a^	100 (0–100)^a^	33 (0–100)	0.026^***^
** Vitality**	45.48 ± 14.16	48.38 ± 18.63	49.50 ± 17.31	47.56 ± 16.61	0.665^*^
** Emotional well-being**	56.25 ± 15.69	58.70 ± 16.21	61.10 ± 12.40	58.36 ± 15.10	0.534^*^
** Social functioning**	50 (25–88)	50 (25–100)	63 (10–100)	50 (10–100)	0.272^***^
** Pain**	38 (0–68)^a^	45 (0–80)	45 (23–70)^a^	45 (0–80)	0.007^***^
** General health**	50 (25–80)	60 (20–85)	57.5 (25–100)	55 (20–100)	0.132^***^
** Health change**	50 (0–100)	25 (0–75)	50 (20–75)	25 (0–100)	0.352^***^

^*^One way ANOVA Test

^**^Chi-square Test

^***^Kruskal Wallis Test (^a^Bonferroni corrected Mann Whitney U Test, *p* < 0.016)

In all three treatment groups, VAS for LBP severity significantly improved with treatment and at one and three months post treatment (*p* < 0.001). There was no significant difference in VAS scores between the treatment groups (*p* > 0.05) (Table 2). MODI scores reduced significantly in all treatment groups (*p* < 0.001) (Table 2). In Group 1, MODI scores continued to significantly reduce at one and three month follow up evaluations when compared to post treatment scores (*p* < 0.001 and *p* = 0.012 respectively). Similarly, significant reduction in MODI scores were also seen in Group 2 at one and three months follow up when compared to post treatment scores (*p* = 0.037 and *p* = 0.045 respectively). On intergroup evaluation, post treatment MODI significantly improved in the control group when compared to the other groups (*p* = 0.006) however this intergroup difference in scores did not continue at one and three month evaluations (Table 2).

**Table 2:** Comparison of within and between group VAS and modified Oswestry scores

	**US group** ***n* = 31**	**KDD group** ***n* = 31**	**Control group** ***n* = 20**	**Between group *p* value**
**Pre treatment VAS score**	7.19 ± 1.60^**a^	6.51 ± 1.72^**a^	6.70 ± 1.49^**a^	0.249^*^
**Post treatment VAS score**	4.25 ± 2.26^a^	4.25 ± 4.20^a^	3.15 ± 1.81^a^	0.379^*^
**1 month post treatment VAS score**	3.96 ± 2.68^a^	3.48 ± 2.04^a^	3.45 ± 1.63^a^	0.621^*^
**3 month post treatment VAS score**	3.41 ± 2.74^a^	3.64 ± 2.21^a^	2.95 ± 1.84^a^	0.588^*^
**Within group p**	< 0.001^**^	< 0.001^**^	< 0.001^**^	
**Pre treatment M.Oswestry**	48.48 ± 18.20^b^	50.19 ± 13.35^b^	40.90 ± 17.16^b^	0.127^*^
**Post treatment M.Oswestry**	36.19 ± 17.00^a,b^	34.80 ± 12.03^a,b^	23.30 ± 13.84^a.b^	0.006^*^
**1 month post treatment M.Oswestry**	25.22 ± 16.92^a^	28.19 ± 17.36^a^	19.70 ± 17.26^a^	0.232^*^
**3 month post treatment M.Oswestry**	26.45 ± 21.90^a^	28.51 ± 16.95^a^	18.60 ± 10.54^a^	0.145^*^
**Within group p**	< 0.001^**^	< 0.001^**^	< 0.001^**^	

^*^One way ANOVA Test

^**^Repeated Meausres ANOVA Test

^a^Post hoc: Paired samples T test

^b^Posthoc:Bonferroni

According to the SF36, physical and social function and pain reduced in all treatment groups following treatment (*p* < 0.05). Role limiation due to physical and emotional problems, emotional well-being, vitality and mental health significantly improved in groups 1 and 2 (*p* < 0.05). However there was no intergroup difference in these values. A significant improvement in general health was recorded in Group 2 (Table 3). Intergroup comparison of SF36 revealed significantly higher physical role, emotional role and pain values in the control group before treatment (*p* = 0.002, *p* = 0.027, *p* = 0.007 respectively); there was no significant difference in these values after treatment. Social function scores were significantly higher in Group 2 when compared to Group 1 three months after treatment (*p* = 0.012) (Table 3).

**Table 3:** Comparison of within and between group SF36 scores

	**US** ***n* = 31**	**KDD** ***n* = 31**	**Control** ***n* = 20**	**Between group** ***p* values**
**Physical functioning**				
**Before treatment**	45.74 ± 21.26^b^	47.09 ± 20.72^b^	59.00 ± 27.65^b^	0.104^*^
**1st month after treatment**	61.51 ± 24.06^b^	66.77 ± 20.02^b^	73.00 ± 17.72^b^	0.172^*^
**3rd month after treatment**	61.61 ± 26.43^b^	70.48 ± 19.20^b^	75.00 ± 17.91^b^	0.087^*^
**Within group p**	0.003^**^	< 0.001^**^	0.036^**^	
**Role -physical**				
**Before treatment**	0 (0–100)^c,d^	0 (0–100)^c,d^	87.5 (0–100)^c^	0.002^***^
**1st month after treatment**	75 (0–100)^d^	100 (0–100)^d^	100 (0–100)	0.114^***^
**3rd month after treatment**	75 (0–100)^d^	100 (0–100)^d^	100 (0–100)	0.063^***^
**Within group p**	< 0.001^****^	< 0.001^****^	0.063^****^	
**Role -emotional**				
**Before treatment**	34.38 ± 39.93^a,b^	47.29 ± 47.75^b^	68.30 ± 39.75^a^	0.027^*^
**1st month after treatment**	64.54 ± 41.24^b^	76.32 ± 39.65^b^	80.00 ± 41.03	0.347^*^
**3rd month after treatment**	63.35 ± 41.70^b^	81.67 ± 35.37^b^	85.00 ± 36.63	0.081^*^
**Within group p**	0.001^**^	0.006^**^	0.110^**^	
**Vitality**				
**Before treatment**	45.48 ± 14.16^b^	48.38 ± 18.63^b^	49.50 ± 17.31	0.576^*^
**1st month after treatment**	54.83 ± 15.62^b^	56.45 ± 21.45^b^	49.75 ± 16.50	0.234^*^
**3rd month after treatment**	55.80 ± 19.58^b^	56.29 ± 23.55^b^	49.00 ± 18.32	0.075^*^
**Within group p**	0.005^*^	0.019^*^	0.957^*^	
**Emotional well-being**				
**Before treatment**	56.25 ± 15.69^b^	58.70 ± 16.21^b^	61.10 ± 12.40	0.382^*^
**1st month after treatment**	63.48 ± 13.13^b^	65.03 ± 16.68	61.30 ± 15.30	0.367^*^
**3rd month after treatment**	62.00 ± 19.34	67.61 ± 17.83^b^	61.15 ± 15.13	0.671^*^
**Within group p**	0.029^*^	0.021^*^	0.996^*^	
**Social functioning**				
**Before treatment**	50 (25–88)^d^	50 (25–100)^d^	63 (10–100)^d^	0.272^***^
**1st month after treatment**	63 (37.5–100)^d^	87.5 (0–100)^d^	87.5 (13–100)^d^	0.221^***^
**3rd month after treatment**	63 (13–100)^c,d^	100 (37.5–100)^c,d^	88 (50–100)^d^	0.012^***^
**Within group p**	0.001^****^	< 0.001^****^	< 0.001^****^	
**Pain**				
**Before treatment**	38 (0–68)^c,d^	45 (0–80)^d^	45 (23–70)^c,d^	0.007^***^
**1st month after treatment**	48 (10–100)^d^	67.5 (35–100)^d^	67.5 (20–90)^d^	0.233^***^
**3rd month after treatment**	58 (10–100)^d^	70 (22.5–100)^d^	63.7 (22.5–90)^d^	0.753^***^
**Within group p**	< 0.001^****^	< 0.001^****^	0.021^****^	
**General health**				
**Before treatment**	50 (25–80)	60 (20–85)^d^	57.5 (25–100)	0.132^***^
**1st month after treatment**	55 (30–80)	65 (15–95)	57.5 (30–100)	0.330^***^
**3rd month after treatment**	55 (25–95)	65 (30–95)^d^	62.5 (30–100)	0.215^***^
**Within group p**	0.248****	0.030^****^	0.590****	
**Mental Health**				
**Before treatment**	38.70 ± 18.07^b^	34.51 ± 15.24^b^	42.25 ± 18.60	0.285^*^
**1st month after treatment**	60.48 ± 26.43^b^	59.67 ± 23.87^b^	52.50 ± 26.77	0.514^*^
**3rd month after treatment**	56.61 ± 30.77^b^	56.41 ± 25.77^b^	57.50 ± 27.02	0.990^*^
**Within group p**	< 0.001^**^	< 0.001^**^	0.099^**^	

^*^One way ANOVA Test (^a^Posthoc: Bonferroni)

^**^Repeated Measures ANOVA Test (^b^Post hoc: Paired samples T test)

^***^Kruskal Wallis Test (^c^Bonferroni corrected Mann Whitney U Test, *p* < 0.016)

^****^Friedman Test (^d^Posthoc: Wilcoxon Signed Ranks Test)

## Discussion

To the best of our knowledge, this is the first prospective randomized control study comparing the efficacy of US and SWD in the treatment of CLBP due to LDH which also evaluates functional outcomes and health concepts pertinent to functional wellbeing. The results of the study show that LBP, disability due to CLBP and physical and social function significantly improved in both those receiving deep heating agents and in the control group. In addition, improvement in disability due to CLBP continued in those treated using deep heating agents for the duration of the three month post treatment follow up period. Psychological wellbeing in the form of role limitation due to emotional problems, emotional well-being, vitality and mental health significantly improved in individuals treated with deep heating agents.

The first clinicial application of therapeutic US in the field of physical therapy began in the 1950s [23]. The thermal effects of US is used to warm tendons, muscle and other tissues and improve blood flow, thus accelerating healing [24]. A recent review on therapeutic US for CLBP by Ebadi et al. concluded that there is some evidence that US may have an effect on improving LBP in the short term, however the certainty of evidence is low [12]. The review also stressed that further high-quality randomized control trials are necessary. Another systematic review on the use of therapeutic US for pain management in CLBP and neck pain, recommended possible use of US as part of a physical modality treatment plan with potential for short term relief of LBP [25]. Once again, paucity of trials and conflicting results, meant US could not be recommended as a monotherapy in the treatment of LBP. Another recent review on the effectiveness of therapeutic US in the management of non-specific CLBP by Haile et al., included a total of six studies and concluded that US can be used to reduce the intensity of non-specific CLBP [26]. Only two of the studies included in the review included mid-term post treatment follow up. The findings of these reviews are in keeping with those in our study as outcomes for CLBP and function in relation to LBP of those receiving US within their treatment plan, was not found to be superior to those receiving cSWD, nor those in the control group. It is important to stress that the disability due to CLBP, as measured using the MODI, continued to improve for the duration of the post treatment follow up period.

Use of SWD as a physical agent is based on the principle of resistance to the passage of electromagnetic energy through tissue which produces an increase in temperature [27, 28]. Continuous SWD provides analgesia through deep heating of tissues, increasing connective tissue elasticity and increasing blood flow to the area of application by encouraging dilation of arterioles and capillaries. Studies on the analgesic effect of SWD in CLBP has become a recent area of interest with several studies still ongoing [29, 30]. Two systematic reviews, published nineteen years apart, which evaluated the evidence of application of SWD in the treatment CLBP both concluded that evidence is limited regarding the effects of SWD in this field, with few articles focusing on pain and most containing varied methodological quality [31, 32]. In contrast, a recent study concluded that cSWD alongside exercise was more effective in reducing pain in patient with CLBP when compared to placebo SWD and pulsed SWD [33]. In the study by Shakoor et al., cSWD was compared to sham cSWD alongside antiinflammatory pharmacological treatment and exercise [34]. Similar to our study, intragroup analysis revealed a significant reduction in LBP in both groups. In contrast to our findings, intergroup analysis revealed significantly better outcomes in the cSWD group. Lack of blinding in the study may have been one factor contributing to biases arising.

The significant improvement in psychological parameters of the SF 36 such as role limitation due to emotional problems, emotional well-being, vitality and mental health, in those receiving deep heating agents, may have been due to their widespread use in the treatment of CLBP in PRM departments across Turkey. Thus, the inclusion of US and cSWD in the treatment protocols of Groups 1 and 2 may have provided the patients with a sense of completeness of therapy which may have been lacking in the control group.

### Limitations

In this study the evaluation of the treatment modalities on subjective pain and functional capacity was limited. Use of additional measures of pain, such as pressure pain thresholds, kinesiophobia and pain catastrophizing would have added value to the study. Similarly, to improve understanding of the effects of these treatments on the functional capactiy of individuals with CLBP, evaluating parameters such as joint range of motion, walking distance and speed, measuring maxium voluntary contraction of muscle groups (for example, lumbar erectors) and muscle strength would have been beneficial.

## Conclusions

The results of this study show that deep heating agents can be used in combination with other electrotherapy modalities and exercise as part of the physical therapy for CLBP in those with LDH and can yield positive mid-term effects. Future studies which utilise further subjective pain measures and measures of functional capacity would provide valuable additional information on the efficacy of various deep heating agents in treating of chronic low back pain in individuals with lumbar disc herniation.

## Data Availability

The datasets used and/or analysed during the current study are available from the corresponding author on reasonable request.
